# Urine 6-Bromotryptophan: Associations with Genetic Variants and Incident End-Stage Kidney Disease

**DOI:** 10.1038/s41598-020-66334-w

**Published:** 2020-06-22

**Authors:** Peggy Sekula, Adrienne Tin, Ulla T. Schultheiss, Seema Baid-Agrawal, Robert P. Mohney, Inga Steinbrenner, Bing Yu, Shengyuan Luo, Eric Boerwinkle, Kai-Uwe Eckardt, Josef Coresh, Morgan E. Grams, Anna Kӧttgen

**Affiliations:** 1 Institute of Genetic Epidemiology, Faculty of Medicine and Medical Center - University of Freiburg, Freiburg, Germany; 2 Department of Epidemiology, Johns Hopkins Bloomberg School of Public Health, Baltimore, MD USA; 3 Welch Center for Prevention, Epidemiology, and Clinical Research, Johns Hopkins University, Baltimore, MD USA; 4 Division of Nephrology, Department of Medicine, Faculty of Medicine and Medical Center - University of Freiburg, Freiburg, Germany; 5 Department of Nephrology and Medical Intensive Care, Charité - Universitätsmedizin Berlin, Berlin, Germany; 6 Department of Nephrology and Transplant Center, Sahlgrenska University Hospital, University of Gothenburg, Gothenburg, Sweden; 7 Metabolon Inc., Durham, NC USA; 8 School of Public Health, The University of Texas Health Science Center at Houston, Houston, USA; 9 Department of Nephrology and Hypertension, Friedrich-Alexander-Universität Erlangen-Nürnberg, 91054 Erlangen, Germany; 10 Division of Nephrology, Johns Hopkins School of Medicine, Baltimore, MD USA; 11 Division of Nephrology, Department of Medicine, University of Mississippi Medical Center, Jackson, MS USA; 12 The Memory Impairment and Neurodegenerative Dementia Center, University of Mississippi Medical Center, Jackson, MS USA

**Keywords:** Prognostic markers, Kidney diseases, Biomarkers, Epidemiology, Genetics research, Outcomes research, Kidney diseases, Risk factors

## Abstract

Higher serum 6-bromotryptophan has been associated with lower risk of chronic kidney disease (CKD) progression, implicating mechanisms beyond renal clearance. We studied genetic determinants of urine 6-bromotryptophan and its association with CKD risk factors and incident end-stage kidney disease (ESKD) in 4,843 participants of the German Chronic Kidney Disease (GCKD) study. 6-bromotryptophan was measured from urine samples using mass spectrometry. Patients with higher levels of urine 6-bromotryptophan had higher baseline estimated glomerular filtration rate (eGFR, p < 0.001). A genome-wide association study of urine 6-bromotryptophan identified two significant loci possibly related to its tubular reabsorption, *SLC6A19*, and its production, *ERO1A*, which was also associated with serum 6-bromotryptophan in an independent study. The association between urine 6-bromotryptophan and time to ESKD was assessed using Cox regression. There were 216 ESKD events after four years of follow-up. Compared with patients with undetectable levels, higher 6-bromotryptophan levels were associated with lower risk of ESKD in models unadjusted and adjusted for ESKD risk factors other than eGFR (<median level: cause-specific hazard ratio [HR] 0.70, 95% confidence interval [CI] 0.51 to 0.97; ≥median level: HR 0.50, 95% CI 0.34 to 0.74). Upon adjustment for baseline eGFR, this association became attenuated, suggesting that urine 6-bromotryptophan may represent a correlated marker of kidney health.

## Introduction

Chronic kidney disease (CKD) is an important global health burden accounting for 1.2 million deaths worldwide in 2015^1,2^. The course of CKD progression is highly variable. Novel biomarkers of CKD progression may lead to improvements in our understanding of the pathophysiology of CKD progression and its prediction^3^. Recently, higher serum 6-bromotryptophan has been associated with lower risk for CKD progression in multiple studies^4^. This molecule is a metabolite formed from the bromination of tryptophan, an essential amino acid, but little is known about its generation, metabolism, or excretion.

Previous studies have shown that higher levels of many blood metabolites are associated with unfavorable renal outcomes and all-cause mortality^5–8^ and thought to reflect reduced renal clearance. It is therefore remarkable that higher serum levels of 6-bromotryptophan were associated with lower risk of CKD progression, implicating mechanisms beyond renal clearance. To learn more about the metabolism of 6-bromotryptophan and its potential handling by the kidney, we carried out a hypothesis-generating genome-wide association study (GWAS) to gain insights into the biological mechanisms influencing 6-bromotryptophan levels in urine in the German Chronic Kidney Disease (GCKD) study, a large contemporary nationwide cohort of CKD patients. Significant loci were validated for association with serum 6-bromotryptophan in an independent cohort of European descent. Among GCKD participants, we also studied the association of urine 6-bromotryptophan with risk factors of CKD and CKD progression.

## Results

**Figure 1:**
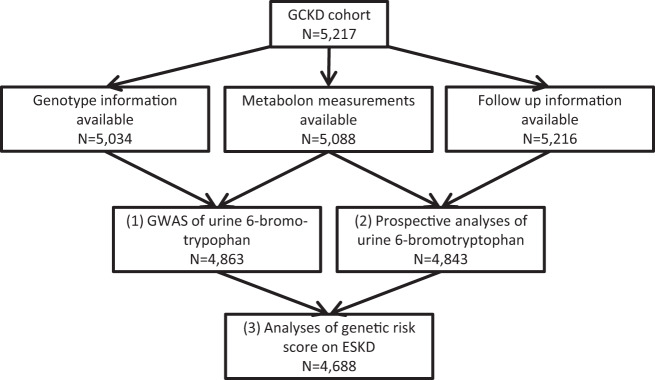
Workflow of analyses in the GCKD study.

The majority of analyses used data from the GCKD study, with the workflow depicted in Fig. 1. Of the 5,217 GCKD participants, 4,843 participants with urine 6-bromotryptophan and incident ESKD information were included in the prospective analysis, and 4,863 participants (including 97% of those in the prospective analysis, n = 4,688) with urine 6-bromotryptophan and genetic data were included in the genome-wide association study.

### Baseline characteristics of GCKD participants

Overall, the mean age of the 4,843 GCKD participants in the prospective analysis was 60 years, and 60% were male (Table 1). Prevalence of diabetes, hypertension, and coronary heart disease was 35%, 96%, and 20%, respectively. In contrast to tryptophan detected in urine of all participants, 6-bromotryptophan was only detectable in 57.3%. The Spearman correlation between tryptophan and 6-bromotryptophan in urine was moderate (r = 0.45).

**Table 1:** Baseline characteristics of the study population (N = 4,843).

Characteristic	Overall	Category of urine 6-bromotryoptophan^a^			
		Undetectable	Low	High	P-value^b^
N	4843	2068	1392	1383	
6-bromotryptophan, mean (SD)	0.96 (0.56)	NA	0.55 (0.17)	1.37 (0.51)	
Leading CKD cause acc. to treating nephrologist, % (n)^c^					0.001
Vascular nephropathy	23 (1107)	21 (443)	23 (321)	25 (343)	
Primary glomerulopathy	19 (912)	18 (381)	19 (268)	19 (263)	
Diabetic nephropathy	15 (704)	16 (340)	16 (217)	11 (147)	
Systemic disease	8 (387)	8 (175)	7 (102)	8 (110)	
Male sex, % (n)	60 (2917)	54 (1108)	61 (856)	69 (953)	<0.001
Age, years, mean (SD)	60.1 (12)	60.6 (11.42)	60.5 (11.89)	58.8 (12.85)	0.001
Smoking, % (n)					<0.001
Non-smoker	41 (1967)	39 (805)	40 (558)	44 (604)	
Former smoker	43 (2102)	44 (900)	43 (597)	44 (605)	
Current smoker	16 (774)	18 (363)	17 (237)	13 (174)	
Education, % (n)					0.020
Higher	17 (806)	15 (314)	17 (240)	18 (252)	
Middle	28 (1343)	27 (551)	28 (396)	29 (396)	
Lower	54 (2595)	56 (1168)	52 (726)	51 (701)	
Other	2 (99)	2 (35)	2 (30)	2 (34)	
Body mass index, kg/m², mean (SD)^d^	29.8 (5.9)	29.7 (6.03)	29.7 (5.8)	29.9 (5.82)	0.718
Waist-to-hip ratio, median (IQR)	0.94 (0.88–1)	0.93 (0.87–0.99)	0.94 (0.89–1)	0.95 (0.89–1.01)	<0.001
Blood pressure, systolic, mmHg, mean (SD)	139.4 (20.3)	138.6 (20.91)	139.1 (20.02)	140.9 (19.59)	0.006
Blood pressure, diastolic, mmHg, mean (SD)	79.3 (11.74)	77.8 (11.76)	79.1 (11.49)	81.7 (11.59)	<0.001
Diabetes mellitus, % (n)	35 (1705)	40 (826)	36 (505)	27 (374)	<0.001
Hypertension, % (n)	96 (4664)	97 (2012)	97 (1353)	94 (1299)	<0.001
Coronary heart disease, % (n)	20 (967)	23 (469)	19 (258)	17 (240)	<0.001
Serum albumin, g/dL, mean (SD)^d^	3.84 (0.44)	3.83 (0.44)	3.81 (0.49)	3.87 (0.40)	0.009
Serum C-reactive protein, mg/L, median (IQR)	2.3 (1.01–4.92)	2.2 (1–4.81)	2.3 (0.98–4.94)	2.3 (1.06–5.1)	0.398
HDL-C, mg/dL, mean (SD)	52 (18.14)	53.4 (19.64)	51.6 (17.07)	50.2 (16.63)	<0.001
LDL-C, mg/dL, mean (SD)	118.3 (43.51)	116.2 (43.47)	119.8 (45.69)	119.7 (41.18)	<0.001
Serum triglycerides, mg/dL, median (IQR)^d^	167.9 (117.54–238.76)	167.6 (116.02–250.66)	170.7 (119.04–235.96)	164.2 (117.64–230.51)	0.226
eGFR, mL/min/1.73 m², mean (SD)	49.4 (18.24)	45 (16.53)	48.7 (16.83)	56.7 (19.71)	<0.001
UACR, mg/g, median (IQR)	50.5 (9.55–384.17)	58.1 (12.26–498.97)	53.3 (8.71–405.42)	37.2 (6.6–265.69)	<0.001
Blood pressure medication: by renin-angiotensin system, % (n)	81 (3924)	82 (1701)	83 (1152)	77 (1071)	<0.001
* ACE inhibitors	47 (2292)	48 (983)	49 (680)	45 (629)	
* ARB	42 (2021)	44 (919)	42 (582)	38 (520)	
* ACE and ARB	8 (389)	10 (201)	8 (110)	6 (78)	
* ACE or ARB	73 (3535)	73 (1500)	75 (1042)	72 (993)	
Diuretic use, % (n)	60 (2926)	71 (1466)	58 (813)	47 (647)	<0.001
* Potassium-sparing	10 (477)	11 (228)	10 (134)	8 (115)	
* Thiazide	26 (1281)	26 (533)	29 (407)	25 (341)	
* Aldosterone antagonist	8 (383)	9 (194)	8 (106)	6 (83)	
* Loop diuretics	38 (1838)	54 (1122)	30 (421)	21 (295)	
Death event, % (n)^e^	7 (325)	8 (167)	6 (78)	6 (80)	
Major causes of death, % (n)^f^					
Due to forgoing of dialysis	0 (9)	0 (4)	0 (2)	0 (3)	
Cardiovascular disease (e.g. myocardial infarction)	2 (108)	3 (60)	2 (25)	2 (23)	
Cerebrovascular disease (e.g. stroke)	0 (14)	0 (6)	0 (4)	0 (4)	
Infection	1 (64)	2 (39)	1 (11)	1 (14)	
ESKD event, % (n)^e^	4 (216)	6 (127)	4(55)	2 (34)	

Abbreviations. HDL-C, high density lipoprotein cholesterol; LDL-C, low density lipoprotein cholesterol; eGFR, estimated glomerular filtration rate; UACR, urine albumin-to-creatinine ratio; ACE, angiotensin converting enzyme; ARB, angiotensin receptor blocker; ESKD, end-stage kidney disease; IQR, interquartile range (25th and 75th percentiles); * Subcategories; ^a^Subcategories according to levels of urine 6-bromotryptophan: undetectable levels; low, range: 0.135 to < 0.84 (median); high, range: 0.84 (median) to 5.31; ^b^Statistical comparison of groups for categorical variables (Pearson’s Chi-squared test) and for continuous variables (Kruskal-Wallis rank sum test); ^c^Major leading causes of CKD as reported by the treating nephrologist (N > 300); ^d^Variables with missing values: body mass index N = 41, serum albumin N = 1, serum triglycerides N = 2 (overall < 1%); ^e^Follow-up time was restricted to the period from study entry to 1,500 days (4.11 years); ^f^Causes of death with at least 50 events, except for death due to forgoing of dialysis.

Across the three groups of 6-bromotryptophan levels (undetectable, low, and high), there were significant differences in kidney function: eGFR (mean levels per group: undetectable 45.0, low 48.7, high 56.7 ml/min/1.73 m^2^, p < 0.001) and UACR (median levels per group: undetectable 58.1, low 53.3, high 37.2 mg/g, p < 0.001). Higher urine 6-bromotryptophan was also significantly associated with lower proportions of prevalent diabetes, hypertension, coronary heart disease, diuretic use, and current smoking status (Table 1).

### Genetic determinants of 6-bromotryptophan levels

**Figure 2:**
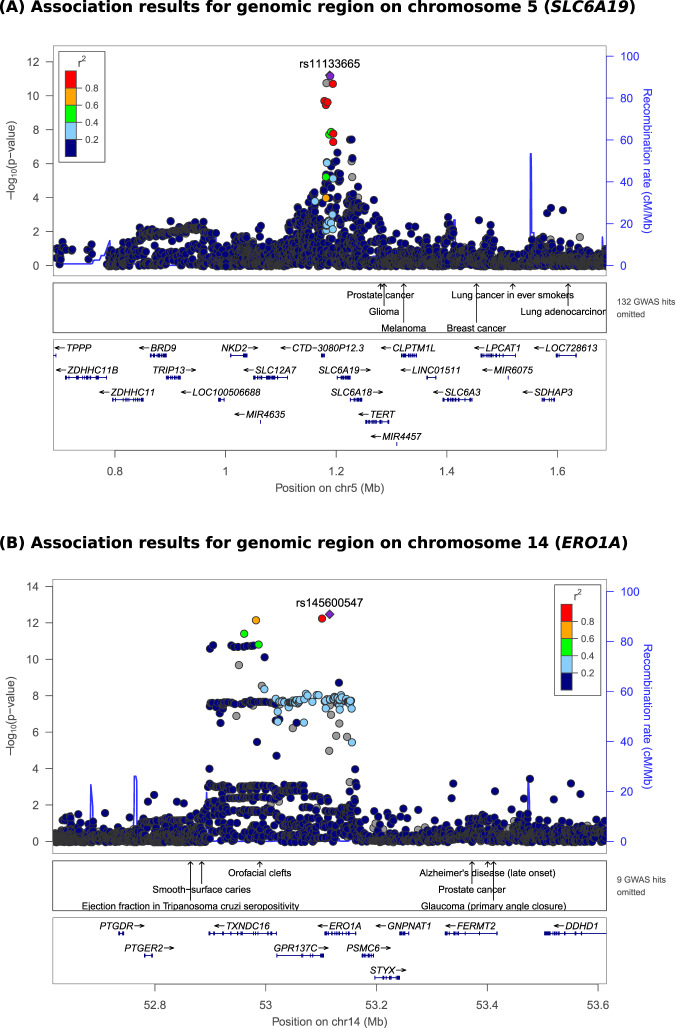
Regional association plots for genome-wide significant loci of urine 6-bromotryptophan in GCKD. Regional association plots for the genome-wide significant loci at chromosome 5 (*SLC6A19*, **A**) and at chromosome 14 (*ERO1A*, **B**). Y-axis: -log_10_(p-value), X-axis: chromosomal location. Marker color corresponds to correlation (r^2^) with the indicated index SNP.

**Figure 3:**
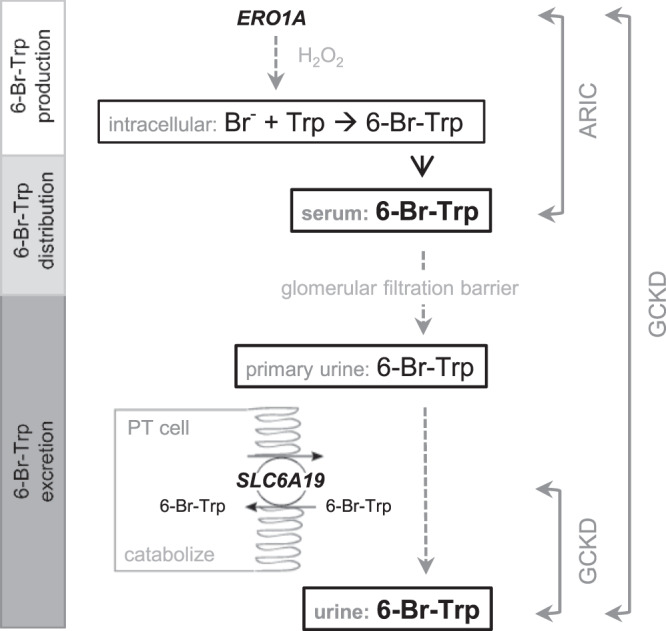
Proposed model of 6-bromotryptophan metabolism based on the findings from this study. Dotted lines and gray font represent hypothesized mechanisms. The genetic association of the *ERO1A* variant with serum (ARIC) and urine 6-bromotryptophan (GCKD) suggests that *ERO1A* may be involved in the production of 6-bromotryptophan. The genetic variant near *SLC6A19* was associated with urine but not serum 6-bromotryptophan, suggesting a mechanism related to reabsorption of 6-bromotryptophan followed by catabolization. SLC6A19 is a plausible biological candidate to mediate this reabsorption. Abbreviations: Br-: bromine; Trp: tryptophan; 6-Br-Trp: 6-bromotryptophan; GCKD: German Chronic Kidney Disease study; ARIC: Atherosclerosis Risk in Communities (ARIC) study.

In the GCKD study, we detected two genome-wide significant loci of urine 6-bromotryptophan. The index SNP on chromosome 5 was rs11133665 (Fig. 2A). This SNP is located upstream of *SLC6A19*, which encodes a renal transport protein known to mediate the reuptake of amino acids such as tryptophan from the urine. It therefore is a plausible candidate for the reuptake of 6-bromotryptophan (Fig. 3). Each copy of the A allele was associated with 11.1% lower urine 6-bromotryptophan levels (p = 6.2 × 10^−12^, Table 2). The other index SNP was rs145600547, an intronic insertion deletion polymorphism in the *ERO1A* (Endoplasmic Reticulum Oxidoreductase 1 Alpha) gene on chromosome 14 (Fig. 2B). Each copy of a CT deletion was associated with 27% lower urine 6-bromotryptophan levels (p = 3.4 × 10^−13^, Table 2). In support, the deletion at rs145600547 was significantly associated with lower *ERO1A* expression in whole blood in the GTEx Project V8 data (p=4.3e-8; https://gtexportal.org/). These two index SNPs together explained 2% of the variance of urine 6-bromotryptophan in GCKD.

**Table 2:** Index SNPs at genome-wide significant loci of urine 6-bromotryptophan in GCKD and their association with serum 6-bromotryptophan in ARIC participants of European ancestry. Abbreviations: GCKD: German Chronic Kidney Disease study; ARIC: Atherosclerosis Risk in Communities (ARIC) study.

Chromosome	5			14		
Gene	*SLC6A19*			*ERO1A*		
Function	upstream			intronic		
Index SNP	rs11133665			rs145600547		
Coded allele/noncoded allele	A/G			A/ACT		
Coded allele frequence	GCKD: 0.28/ARIC: 0.28			GCKD: 0.03/ARIC: 0.03		
**Association with 6-bromo-tryptophan in …**	Effect in %*	Beta* (SE)	**P-value**	Effect in %*	Beta* (SE)	**P-value**
urine (GCKD, N = 4863)	−11.1	−0.17 (0.02)	**6.2E-12**	−27.0	−0.45 (0.06)	**3.4E-13**
serum (ARIC, N = 1433)	1.2	0.02 (0.01)	7.5E-02	−11.1	−0.17 (0.03)	**1.9E-09**

*beta on the log_2_ scale and effect in % = (2^beta^ -1)*100.

Imputation quality: rs11133665, GCKD 1.0, ARIC 0.99; rs145600654, GCKD 0.93, ARIC 0.76 (info from impute2 output).

rs11133665 function based on UCSC, downstream gene is CTD-3080P12.3.

We next tested whether these two index SNPs were associated with serum 6-bromotryptophan levels among participants of European descent in the Atherosclerosis Risk in Communities (ARIC) study, a population-based cohort with a mean age of 55 and mean eGFR of 98 mL/min/1.73 m^2^ (Supplementary Table 1). Whereas rs11133665, upstream of *SLC6A19*, did not show a significant association with serum 6-bromotryptophan levels (p = 0.07), rs145600547 at *ERO1A* was significantly associated in a direction consistent with its effect on urine 6-bromotryptophan (each CT deletion was associated with 11.1% lower serum levels, p = 1.9 × 10^−9^, Table 2) and had the strongest association in the entire region (Supplementary Fig. 1). The association at the *SLC6A19* locus with urine but not serum 6-bromotryptophan is consistent with the reuptake of filtered 6-bromotryptophan by the SLC6A19 transporter at the apical membrane of tubular epithelial cells that subsequently does not exit the cell on the basolateral side into the blood, for example, due to intracellular catabolism (Fig. 3). In contrast, the association with 6-bromotryptophan at *ERO1A* was observed in blood and may reflect mechanisms related to the production of 6-bromotryptophan rather than its reuptake (Fig. 3).

### Association between urine 6-bromotrytophan and ESKD

**Figure 4:**
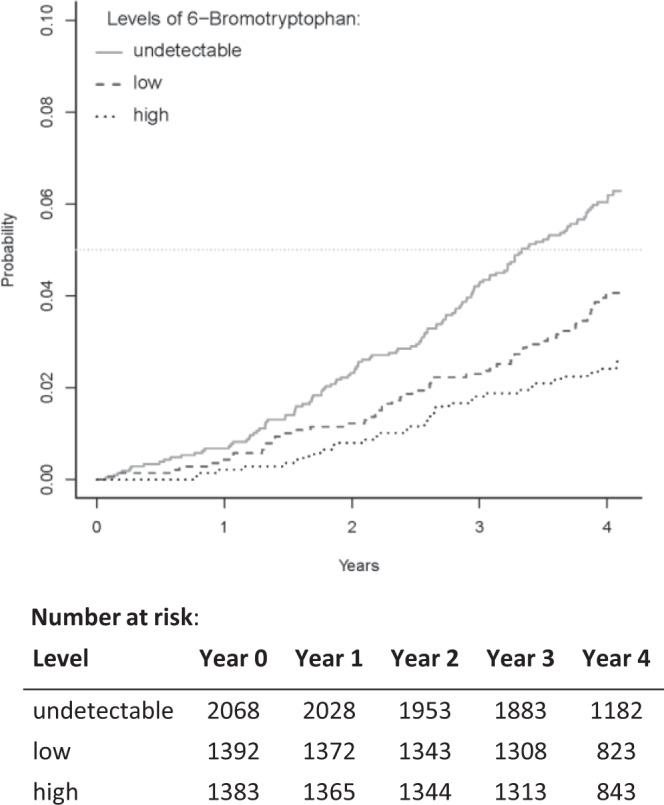
Cumulative incidence function of ESKD events by the three categories of urine 6-bromotrytophan levels. Categories of urine 6-bromotryptophan levels: (1) undetectable: patients with undetectable levels; (2) low: patients with levels < median detected level (0.84); (3) high: patients with levels ≥0.84.

Among participants in the GCKD study, there were 216 ESKD events after four years of follow-up (median [range] of follow-up in years: 4.04 [0.06–4.11]). Compared with those having undetectable urine 6-bromotryptophan levels, those with low and high levels had lower risk of ESKD in unadjusted analysis, age- and sex-adjusted analyses, and with additional adjustment for baseline non-kidney risk factors (Fig. 4, Table 3). For example, compared to those with undetectable levels, those with high urine 6-bromotryphan levels had a cause-specific hazard ratio (HR) of 0.39 (95% CI: 0.26 to 0.58) after adjustment for age, sex, and non-kidney risk factors (Model 3, see Methods for selection of risk factors). With the addition of baseline UACR as a covariate, the cause-specific hazard ratios were somewhat attenuated but remained significant (Model 4). A sensitivity analysis that categorized urine 6-bromotryptophan levels into five groups yielded very similar results (Supplementary Table 2), with no indication of departure from linearity. With the addition of baseline eGFR, the association between urine 6-bromotryptophan and ESKD was no longer significant (Model 5, low group HR: 0.98, 95% CI: 0.71 to 1.36; high group HR: 1.25, 95% CI: 0.83 to 1.89, Table 3). In the competing risk analysis using death not due to forgoing dialysis and without ESKD previously as the competing event, urine 6-bromotryptophan was also not significantly associated with ESKD in Model 5 (Supplementary Table 3).

**Table 3:** Association between urine 6-bromotryptophan and incident ESKD.

Category^a^:	Cause-specific hazard ratio (95% confidence interval)		
	Undetectable	Low	High
Model 1	1.00	**0.63 (0.46, 0.86)**	**0.39 (0.27, 0.57)**
Model 2	1.00	**0.59 (0.43, 0.81)**	**0.33 (0.23, 0.49)**
Model 3	1.00	**0.64 (0.46, 0.88)**	**0.39 (0.26, 0.58)**
Model 4	1.00	**0.70 (0.51, 0.97)**	**0.50 (0.34, 0.74)**
Model 5	1.00	0.98 (0.71, 1.36)	1.25 (0.83, 1.89)

N = 4,843, N_events_ = 216.

^a^Subcategories according to levels of urine 6-bromotryptophan: undetectable levels; low: range: 0.135 to <0.84 (median); high: range: 0.84 (median) to 5.31.

Covariate adjustment:

Model 1: unadjusted.

Model 2: age + sex.

Model 3: Model 2 + smoking + waist-to-hip ratio + diastolic blood pressure + prevalent diabetes + coronary heart disease + high density lipoprotein cholesterol + low density lipoprotein cholesterol + diuretic use.

Model 4: Model 3 + urine albumin-to-creatinine ratio.

Model 5: Model 4 + eGFR.

The genetic score summarizing a genetic effect on higher urine 6-bromotryptophan levels showed cause-specific hazard ratios in the same direction, but was not significantly associated with ESKD (N = 4,688, 209 events, HR: 0.73, 95% CI: 0.32, 1.69, p = 0.46).

Lastly, we evaluated the addition of urine 6-bromotryptophan to the Tangri 4-variable kidney failure risk model, a widely-validated model^9,10^. This addition led to a negligible increase in the C-statistic (0.8526 vs 0.8531), and a likelihood ratio test comparing both models did not reveal any significant difference (p = 0.74). These results indicate that urine 6-bromotryptophan does not contribute to risk prediction beyond the variables included in the Tangri model.

## Discussion

We detected two genetic loci of urine 6-bromotryptophan levels providing insights into its metabolism: while *SLC6A19* encodes a transporter and likely responsible for its tubular reuptake, the signals at the *ERO1A* locus may reflect its generation. Among GCKD participants, 6-bromotryptophan was detected in urine in just over half of the population. Compared to those with undetectable levels, those with detectable levels of urine 6-bromotryptophan had higher kidney function and lower prevalence of risk factors of ESKD. In prospective analyses, higher urine 6-bromotryptophan levels were significantly associated with lower risk of ESKD in models that were not adjusted for eGFR and did not have significant association after adjusting for eGFR.

Previously, higher serum 6-bromotryptophan levels were reported to be associated with lower risk of CKD progression in three cohorts: the African American Study of Kidney Disease and Hypertension (AASK), Bio*Me*, and the Modification of Diet in Renal Disease (MDRD) study^4^. Associations were robust after adjustment for eGFR and albuminuria as well as other kidney disease risk factors. In the present study, the direction of association between urine 6-bromotryptophan and ESKD was consistent with the reported association of higher serum 6-bromotryptophan with higher kidney function and lower risk of CKD progression, but the results were not independent of eGFR. This suggests that the mechanisms related to its tubular reuptake may be correlated with eGFR.

Whereas urine 6-bromotryptophan was detected in 57% of the GCKD participants, serum 6-bromotryptophan was detected in the overwhelming majority of the participants in other studies, including the ARIC study^4^. This is consistent with free filtration of this metabolite, followed by its tubular reabsorption, for which we detected SLC6A19 as a candidate transporter in an unbiased genome-wide screen. This finding is biologically plausible: rs11133665, the index SNP at *SLC6A19*, has been identified in a previous GWAS of urinary metabolites, with the A allele associated with lower levels of tyrosine and histidine^11^, directionally consistent with its association with lower urine 6-bromotryptophan and reflecting more efficient reuptake of amino acids. Moreover the associations of *SLC6A19* variants with urine tyrosine and histidine levels were not observed in blood^11^, consistent with the lack of significant association at the *SLC6A19* locus for serum 6-bromotryptophan. S*LC6A19* encodes a transporter of amino acids at the apical membrane of tubular epithelial cells^12^. Rare mutations in S*LC6A19* cause autosomal-recessive Hartnup disorder^13^, a disease featuring the loss of amino acids in urine, including tryptophan^14^. The mouse model of Hartnup disorder shows elevated concentrations of many neutral amino acids in urine, while plasma concentrations were normal^15^, which is consistent with our observation of genetic associations with urine but not serum 6-bromotryptophan levels.

Little is known about the generation of 6-bromotryptophan; it is a metabolite formed from the bromination of tryptophan but the localization of this reaction is unknown. Bromine, a trace element^16^, was shown to be an essential co-factor for the formation of sulfilimine, a crosslink protein in the assembly of collagen IV scaffolds^17^. This reaction involves peroxidasin, a heme peroxidase, hydrogen peroxide, and bromide, the ionic form of bromine, to generate hypobromous acid^17,18^. Here, the other genetic locus detected in the GWAS of 6-bromotryptophan levels may provide novel insights: the identified insertion deletion polymorphism rs145600547 maps into an intron of *ERO1A* and had the strongest association with urine 6-bromotryptophan in GCKD and with serum 6-bromotryptophan in ARIC. The oxidoreductase encoded by *ERO1A* plays a role in disulfide bond formation, with reactive oxygen species such as hydrogen peroxide generated as part of this pathway. It is therefore tempting to speculate that *ERO1A* is involved in the generation of hydrogen peroxide required for the bromination of tryptophan^19^, and this hypothesis would also be consistent with the observed association of the *ERO1A* locus on both urine and blood 6-bromotryptophan levels.

We observed that adjustment for eGFR led to an attenuation of the association between urine 6-bromotryptophan levels with ESKD, whereas this was not the case with serum 6-bromotryptophan in previous studies of CKD patients. A potential explanation is that urine levels of 6-bromotryptophan reflect processes that are correlated with eGFR more strongly than those of serum 6-bromotryptophan. If urine 6-bromotryptophan and eGFR levels show stronger correlations than serum 6-bromotryptophan and eGFR, for example because tubular reabsorptive capacity is correlated with both eGFR and urine 6-bromotryptophan levels, this could explain the attenuation of the association of urine 6-bromotryptophan and ESKD risk upon adjustment for eGFR. In agreement, our genetic studies support that tubular re-uptake of the metabolite does not influence its blood levels, making this a plausible explanation for differences to previous studies of serum 6-bromotryptophan levels. Alternatively or in addition, a more sensitive assay for very low levels of 6-bromotrypotophan in urine (e.g., a targeted MS assay) might allow for better discrimination of ESKD risk. Lastly, other differences between the GCKD study and previous studies, such as the proportion of individuals with diabetes, may also contribute to the observed differences. In any case, our study suggests that urine 6-bromotryptophan represents an interesting novel biomarker of ESKD risk, even if it does not improve ESKD prediction in CKD patients beyond eGFR. In contrast to established markers of kidney function such as creatinine and urea, where higher levels reflect poor kidney function, higher levels of both serum and urine 6-bromotryptophan levels were associated with better eGFR and lower levels of markers of kidney damage. These results support the notion that 6-bromotryptophan levels may mark an essential protective process in the kidney.

Our study has several strengths. It was conducted in a large contemporary cohort of patients with CKD with rich phenotypic characterization and over 4 years of follow-up in whom urine 6-bromotryptophan was measured and adjusted for urine dilution based on a probabilistic quotient derived from a large number of metabolites. The two genome-wide significant loci of 6-bromotryptophan provide additional insight on the metabolism of 6-bromotryptophan with direct relevance in humans. However, the modest effects of the genetic variants on urine 6-bromotryptophan levels limited statistical power to use a genetic score to investigate a potential causal relationship between 6-bromotryptophan and ESKD. Other limitations include levels of urine 6-bromotryptophan were measured from spot urine with relative quantification, and information on dietary factors that may influence 6-bromotryptophan levels were not available. The GCKD study does not have measures of serum 6-bromotryptophan for direct comparison with urine 6-bromotryptophan for risk association and the estimation of fractional excretion. In addition, information on eGFR slopes over time is not yet available for an analysis of changes in kidney function, as creatinine collection at follow-up visits is still ongoing in the GCKD study.

In conclusion, this study extends previous work on the association between higher serum 6-bromotryptophan and lower risk of CKD progression by investigating the association between urine 6-bromotryptophan and ESKD in a large cohort of CKD patients. We show that urine 6-bromotryptophan is detectable in approximately half of the patients and has strong associations with markers of baseline kidney function and damage. The strong association with ESKD is not independent of eGFR. Our study shows how integration of genetic information with the evaluation of novel biomarkers of kidney disease yields novel insights into their metabolism, and supports the notion that higher 6-bromotryptophan may mark a protective process with respect to kidney function that is different from filtration and worth further investigation.

## Materials and Methods

### German Chronic Kidney Disease (GCKD) study

The GCKD study is a prospective cohort study in Germany that enrolled 5,217 adult patients of European descent under regular care of nephrologists^20^. Main eligibility criteria were eGFR of 30–60 mL/min/1.73 m^2^ or eGFR > 60 mL/min/1.73 m^2^ with urinary albumin-to-creatinine ratio (UACR) > 300 mg/g. All participants provided written informed consent^21^. The GCKD study was approved by local ethics committees of each of the nine participating German institutions reported in the Supplementary Note and is registered in the German national registry for clinical studies (DRKS 00003971). The study is conducted according to national laws and relevant guidelines.

All patients underwent standardized, questionnaire-based interviews and physical examinations by trained personnel. Biosamples collected were immediately processed and shipped on dry ice to a central biobank where they were stored at −80 °C for future usage^22^. Measurement and definitions of baseline characteristics used in this project are described in the Supplementary Methods section. These variables were selected a priori to include study-specific characteristics, diagnostic measures of CKD as well as known CKD risk factors^23^. End-stage kidney disease (ESKD) and death including the cause of death, were obtained from dialysis protocols, hospital discharge records, and death certificates collected at yearly follow-up visits, and systematically abstracted by physicians on a continuous basis.

#### Measurement of urine 6-bromotryptophan

6-bromotryptophan was measured from spot urine samples collected at the GCKD enrollment visit. 6-bromotryptophan was measured by Metabolon, Inc (Morrisville, NC)^4^. Details of the methods for metabolite profiling are reported in the Supplementary Methods section. Briefly, 6-bromotryptophan was quantified using reverse phase ultra-high performance liquid chromatography-tandem mass spectrometry (UPLC-MS/MS^n^) methods and negative ion mode electrospray ionization^24^ and identified by matching detected features to an in-house spectral and chromatographic library of authentic reference standards containing the retention time/index (RI), mass to charge ratio (m/z), and MS/MS fragmentation data. The levels were quantified using area-under-the-curve of the MS peaks following inter-day normalization. After quality control, measurements for 5,088 GCKD patients were available for analyses. 6-bromotryptophan was detected in 57% (n = 2,909) of the participants, with missingness likely due to values being below the limit of detection of the untargeted metabolite profiling approach^25^. We evaluated potential reasons for missingness but did not find a link, for instance to enrollment year (Supplementary Fig. 2A,B). To account for urine concentration, we applied the probabilistic quotient normalization method, which uses the median values of all metabolites with complete measurements as reference and was found to have better performance in CKD patients than other methods that use a single substance, such as urine creatinine^26,27^.

#### Imputation and genome-wide association analysis of urine 6-bromotryptophan

Detailed information on genotyping and imputation based on the 1000 Genomes Project ALL haplotypes – Phase 3^28^ integrated variant set using SHAPEIT v2^29^ and IMPUTE2 (v2.3.1)^30^ in the GCKD study was reported previously^27^. Filtering for imputation quality (info) value of >0.8 and minor allele frequency (MAF) ≥ 1% yielded 9,281,895 high-quality imputed markers.

Given the limit of detection is a major contributing factor to missing values in the untargeted metabolite approach^25^, missing values of urine 6-bromotryptophan were imputed to the minimum observed measurement before correction for urine dilution using the probabilistic quotient normalization method for the GWAS in order to increase statistical power. Measurements were log_2_-transformed resulting in a near normal distribution (Supplementary Fig. 2C). Linear regression was conducted using SNPtest v2.5.2^31^ adjusting for age, sex, the first three genetic principal components, log-transformed eGFR and UACR. The GWAS included 4,863 participants with imputed genotype dosage, urine 6-bromotryptophan and covariate information (Fig. 1). The significance threshold for the GWAS was set at 5*10^−8^. The quantile-quantile plot and Manhattan plot of the results are presented in Supplementary Fig. 3, and indicated no major sources of unmodeled stratification. Regional association plots were generated by LocusZoom (v1.3)^32^ using genotypes from the GCKD study to calculate linkage disequilibrium.

#### Association analysis of baseline characteristics and ESKD with urine 6-bromotryptophan

Given the limit of detection is a major contributing factor to missing values in the untargeted metabolite approach^25^, we categorized 6-bromotryptophan levels into three groups: undetectable (43%), low (<median among those with detectable levels) and high (≥median). Separating the detectable levels at the median allows for some inspection of linearity of effects.

Baseline characteristics of the participants were analyzed by these three groups of 6-bromotryptophan levels using chi-square-test for categorical variables and Kruskal-Wallis rank sum test for continuous variables.

ESKD events were defined as a composite endpoint of incident dialysis or kidney transplantation (N = 210) and death due to forgoing of dialysis (N = 6). To evaluate the association between urine 6-bromotryptophan categories and ESKD, we used cause-specific Cox regression models. Patients without an event were censored at the time of last visit or the time of death for reasons not attributable to kidney disease. We evaluated the association between urine 6-bromotryptophan categories and ESKD first using an unadjusted model (Model 1) and successively adding risk factors of CKD. Given that baseline UACR and eGFR are the two most important predictors of CKD progression^9^, these two variables were added last. Model 2 adjusted for age and sex. Model 3 added baseline non-kidney risk factors: smoking, waist-to-hip ratio, diastolic blood pressure, diabetes, coronary heart disease, high density lipoprotein cholesterol (HDL-C), low density lipoprotein cholesterol (LDL-C), and diuretic use. These factors were selected based on significantly association with urine 6-bromotryptophan categories in univariate analysis (Table 1, p < 0.001) and excluding categorical variables with >90% of the participants in one of its categories. Model 4 added log(UACR), and Model 5 added log(eGFR). We examined Schoenfeld residuals to assess the proportional hazards assumption, and found no violations (data not shown). The competing event, death from non-ESKD causes (N = 273), was investigated similarly. Reported analyses were restricted to complete cases (N = 4,843, Fig. 1).

In addition, a genetic score was calculated as the sum of the alleles of the index SNPs associated with higher urine 6-bromotryptophan in the GWAS of urine 6-bromotryptophan, weighted by respective effect estimates. We assessed the association between the genetic score and ESKD using cause-specific Cox regression controlling for age and sex among 4,688 GCKD participants with both genetic and prospective data (Fig. 1).

### Sensitivity analysis

We conducted sensitivity analyses to investigate other factors that might influence the association between urine 6-bromotryptophan and kidney function: potential non-linear relations between urine 6-bromotryptophan and baseline kidney function. We inspected scatter plots of urine 6-bromotryptophan levels against baseline eGFR and UACR (Supplementary Fig. 4). For the prospective analysis of ESKD, we categorized urine 6-bromotryptophan levels into five groups: undetectable, and four groups by quartiles of the detectable values and reassessed the association between urine 6-bromotryptophan and ESKD using the Cox regression models described above. A trend test that coded the five groups as a numeric variable was used as a test for linearity.

To evaluate the extent to which urine 6-bromotryptophan might improve ESKD prediction, we compared goodness of fit measures of the Tangri 4-variable kidney failure risk model, a widely-validated model^9,10^, with the same model adding urine 6-bromotryptophan as a 3-category predictor in the GCKD study.

### ARIC study

To determine whether genetic loci significantly associated with urine 6-bromotryptophan levels in GCKD were also associated with serum 6-bromotryptophan, we tested the association of the index SNPs with serum 6-bromotryptophan among participants of European descent in the ARIC study (N = 1,433, Supplementary Table 1). This study was approved by Institutional Review Board of the Johns Hopkins University. Participants included in this study have provided informed consent for genetic studies. The ARIC study is conducted according to national laws and relevant guidelines.

Details of genotyping and imputation in the ARIC study are reported in the Supplementary Methods. Details of the metabolite profiling in the ARIC study were reported previously^33^. Briefly, metabolite profiling of 2,153 participants (1,553 European descent and 600 African Americans) was performed using fasting serum samples that had been stored at −80 °C since collection in 1987–1989. In total, 1,160 known and unknown metabolites were quantified by Metabolon Inc. (Durham, USA) using untargeted, gas chromatography-mass spectrometry and liquid chromatography-mass spectrometry (GC-MS and LC-MS)^34^. Serum 6-bromotryptophan was detected in 97.4% of the samples (1,510 European ancestry and 585 African Americans).

In the analysis, we performed linear regression using natural log-transformed serum 6-bromotryphan as the outcome adjusting for age, sex, study-center, and ten genetic principal components. The effect estimates were converted to log_2_ scale for reporting to match the transformation of urine 6-bromotryptophan in GCKD. The significance threshold was set at 0.05 divided by the number of tested index SNPs.

## Supplementary information

Supplementary information.

## Data Availability

With respect to GCKD, public posting of individual level participant data is not covered by the informed patient consent. As stated in the patient consent form and approved by the Ethics Committees, pseudonymized datasets can be obtained by collaborating scientists upon approval of a scientific project proposal by the steering committee of the GCKD study: www.gckd.org. The ARIC study genetic data is available at dbGaP (accession: phs000090). The metabolite data is available upon approval of a manuscript proposal by the ARIC Publication Committee as described in https://sites.cscc.unc.edu/aric/distribution-agreements.
