# Supplementary material for: Urine 6-Bromotryptophan: Associations with Genetic Variants and Incident End-Stage Kidney Disease
Source: Sci Rep. 2020 Jun 22;10:10018. doi: 10.1038/s41598-020-66334-w (PMC7308283; doi:10.1038/s41598-020-66334-w)

**Urine 6-Bromotryptophan: Associations with Genetic Variants and**

**Incident End-Stage Kidney Disease**

Peggy Sekula; Adrienne Tin; Ulla T. Schultheiss; Seema Baid-Agrawal; Robert P. Mohney; Inga Steinbrenner; Bing Yu; Shengyuan Luo; Eric Boerwinkle; Kai-Uwe Eckardt; Josef Coresh; Morgan E. Grams; Anna Kӧttgen

**Supplementary Material**

**Supplementary Methods:**

1. Measurement and definitions of GCKD baseline characteristics
2. Metabolite measurements in GCKD
3. Genotyping and imputation in ARIC

References

**Supplementary Note:** List of GCKD Participating Institutions and Investigators

**Supplementary Table 1:** ARIC – population characteristics

**Supplementary Table 2**: Sensitivity analysis on the association between urine 6-bromotryptophan and incident ESKD by categorizing urine 6-bromotryptophan levels into five groups

**Supplementary Table 3**: Association between urine 6-bromotryptophan and death for any reason except due to forgoing dialysis

**Supplementary Table 4:** Summary of properties of urine 6-bromotryptophan and its measurement in the GCKD study

**Supplementary Figure 1:** Regional association plot of the *ERO1A* locus in the ARIC study

**Supplementary Figure 2:** Distribution of urine 6-bromotryptophan measurements in GCKD

**Supplementary Figure 3:** Q-Q plot and Manhattan plot of the GWAS of urine 6-bromotryptophan in the GCKD cohort

**Supplementary Figure 4:** Plots of urine 6-bromotryptophan levels in relation to baseline kidney function markers

**Supplementary Methods**

***1. Measurements and definitions of GCKD baseline characteristics***

Measurements and definition of baseline characteristics used in this project:

Age, sex, smoking, and education were based on self-report at the enrollment visit. The leading cause of CKD was provided by the treating nephrologist based on clinical information. Medication intake was systematically obtained for all GCKD patients and coded using the Anatomical Therapeutic Chemical (ATC) classification system. Systolic and diastolic blood pressures were the mean of three measures taken in an upright sitting position after five minutes of rest using a standardized device (Omron M5 Professional devices). Hypertension was defined as the use of blood pressure lowering medications, systolic blood pressure ≥140 mm Hg, or diastolic blood pressure ≥90 mm Hg. Diabetes mellitus was defined as the use of antidiabetic medications or hemoglobin A1c levels ≥6.5%. Coronary artery disease was defined as having a history of percutaneous coronary intervention, bypass surgery, or myocardial infarction. From stored serum samples, C-reactive protein (CRP) was measured by CRPHS assay, HDL by HDL-C plus 3^rd^ generation assay and LDL by LDL-C plus 2^nd^ generation assay (Roche). eGFR was calculated from serum creatinine (IDMS traceable enzymatic assay Creatinine plus, Roche) using the Chronic Kidney Disease Epidemiology Collaboration (CKD-EPI) equation.[^1^](#_ENREF_1) UACR was measured from spot urine (creatinine: IDMS traceable enzymatic assay Creatinine plus, Roche; albumin: ALBU-XS assay, Roche).

***2. Metabolite measurements in GCKD***

For the measurement of metabolites, urine specimens collected at baseline were sent in three batches for non-targeted MS analysis to Metabolon, Inc. Sample preparation was carried out as described previously.[^2^](#_ENREF_2)^,^[^3^](#_ENREF_3) Briefly, recovery standards were added prior to the first step in the extraction process for quality control purposes. To remove protein, dissociate small molecules bound to protein or trapped in the precipitated protein matrix, and to recover chemically diverse metabolites, proteins were precipitated with methanol under vigorous shaking for 2 min (Glen Mills Genogrinder 2000) followed by centrifugation. The resulting extract was divided into fractions and vacuum dried. For each sample, dried extracts were dissolved in injection solvent containing eight or more injection standards at fixed concentrations, depending on the platform, to assure injection and chromatographic consistency.

Each sample was analyzed by four ultra-high performance liquid chromatography-tandem mass spectrometry (UPLC-MS/MS) methods:[^4^](#_ENREF_4) 1) One aliquot was analyzed using acidic positive ion conditions, chromatographically optimized for more hydrophilic compounds. In this method, the extract was gradient eluted from a C18 column (Waters UPLC BEH C18-2.1x100 mm, 1.7 μm) using water and methanol, containing 0.05% perfluoropentanoic acid (PFPA) and 0.1% formic acid (FA). 2) A second aliquot was also analyzed using acidic positive ion conditions; however, it was chromatographically optimized for more hydrophobic compounds. In this method, the extract was gradient eluted from the same aforementioned C18 column using methanol, acetonitrile, water, 0.05% PFPA and 0.01% FA and was operated at an overall higher organic content. 3) A third aliquot was analyzed using basic negative ion optimized conditions using a separate dedicated C18 column. The basic extracts were gradient eluted from the column using methanol and water, however with 6.5mM Ammonium Bicarbonate at pH 8. 4) The fourth aliquot was analyzed via negative ionization following elution from a HILIC column (Waters UPLC BEH Amide 2.1x150 mm, 1.7 μm) using a gradient consisting of water and acetonitrile with 10mM Ammonium Formate, pH 10.8. 5). A fifth aliquot was reserved for backup.

Three types of controls were analyzed in concert with the experimental samples:[^5^](#_ENREF_5) Samples generated from a pool of human urine extensively characterized by Metabolon, Inc. served as technical replicate throughout the data set; extracted water samples served as process blanks; and a cocktail of standards spiked into every analyzed sample allowed instrument performance monitoring. Instrument variability was determined by calculating the median relative standard deviation (RSD) for the standards that were added to each sample prior to injection into the mass spectrometers (median RSD=5-7%; n=31 standards). Overall process variability was determined by calculating the median RSD for all endogenous metabolites (i.e., non-instrument standards) present in 100% of the pooled human urine samples (median RSD=7-9%; n>1,000 metabolites).

Experimental samples and controls were randomized across the platform run.[^5^](#_ENREF_5)^,^[^6^](#_ENREF_6) All methods utilized a Waters ACQUITY UPLC and a Thermo Scientific Q-Exactive high resolution/accurate mass spectrometer interfaced with a heated electrospray ionization (HESI-II) source and Orbitrap mass analyzer operated at 35,000 mass resolution. Instruments were tuned and calibrated for mass resolution and mass accuracy daily. The MS analysis alternated between MS and data-dependent MSn scans using dynamic exclusion. The scan range varied slighted between methods but covered 70-1,000 m/z.

Metabolites were identified by automated comparison of the ion features in the experimental samples to a reference library of chemical standard entries that included retention time, molecular weight (m/z), preferred adducts, and in-source fragments as well as associated MS spectra, and curated by visual inspection for quality control using software developed at Metabolon.[^7^](#_ENREF_7)^,^[^8^](#_ENREF_8) Identification of known chemical entities was based on comparison to Metabolon’s spectral library of >4,500 purified chemical standards. Commercially available purified standard compounds have been acquired and registered into LIMS for distribution to the various UPLC-MS/MS platforms for determination of their detectable characteristics.[^5^](#_ENREF_5) Known metabolites reported in this study conformed to confidence Level 1 (the highest confidence level of identification) of the Metabolomics Standards Initiative,[^9^](#_ENREF_9)^,^[^10^](#_ENREF_10) unless otherwise denoted with an asterisk. Additional mass spectral entries have been created for structurally unnamed biochemicals (>2,750 in the Metabolon library), which have been identified by virtue of their recurrent nature (both chromatographic and mass spectral). These compounds have the potential to be identified by future acquisition of a matching purified standard or by classical structural analysis and were included in this study.

Peaks were quantified using area-under-the-curve.[^8^](#_ENREF_8) Raw area counts for each metabolite in each sample were normalized to correct for variation resulting from instrument inter-day tuning differences by the median value for each run-day, therefore, setting the medians to 1.0 for each run. This preserved variation between samples but allowed metabolites of widely different raw peak areas to be compared on a similar graphical scale.

After receipt of the quantified metabolites from Metabolon, Inc, an in-house pipeline was set up for data quality control, filtering of metabolites and samples, and for normalizing concentrations to account for urine dilution. No sample had to be excluded for a high proportion of missing data (>50%). Due to suspected duplication, 8 samples were excluded. 45 metabolites were excluded as they were detectable in less than 20 samples. To account for differences in urine dilution, the probabilistic quotient was computed based on endogenous metabolites with <1% missing values.[^11^](#_ENREF_11) None of the remaining metabolites was excluded due to low variance (<0.01) or many outliers (>5% of samples in >5 standard deviations [SD]). One sample was excluded as it was > 5 SDs in one of the first 15 principal components of metabolites without initial missing values. In the end, measurements of 1,487 metabolites from 5,088 urine samples were available for subsequent analyses. Of the respective 5,088 GCKD participants, genotype data was also available for 4,912 participants.

In this project, measurements of 6-bromotryptophan were extracted for analysis. Information on biochemical properties of 6-bromotryptophan is reported in **Supplementary Table 4**. 6-bromotryptophan was detected in 57% of the participants, with missingness likely due to values being below the limit of detection of the untargeted metabolite profiling approach.[^12^](#_ENREF_12) For the analysis with prospective endpoints, measurements were categorized into the three groups of 1) undetectable, 2) low (< median), and 3) high (≥ median). For the genome-wide association study of 6-bromotryptophan, missing values were imputed with the minimum observed measurement before concentrations were corrected for the differences in urine dilution. Furthermore, measurements were log_2_-transformed resulting in a near normal distribution (**Supplementary Figure 1C**).

***3. Genotyping and imputation in ARIC***

The genotyping in European-American participants used the Affymetrix 6.0 platform and was supported by the National Institutes of Health Gene Environment Association Studies (GENEVA) project.[^13^](#_ENREF_13)^,^[^14^](#_ENREF_14) The initial quality filters included a call rate of 0.95, check for blind duplicates in each plate and gender mismatch. Of the 910,030 SNPs genotyped, 841,820 passed the initial quality filters. Of the SNPs that failed the initial quality filters, 96% were due to the call rate. After the genotype data of 9,713 individuals was received from GENEVA, additional quality filters were applied to exclude individuals who were more than 8 SD away from any of the 10 genetic principal components or closely related based on identity by state distance (DST >0.86) calculated by PLINK.[^15^](#_ENREF_15) Altogether 658 individuals were excluded resulting in a genotype dataset of 9055 individuals.

Genotype dosage imputation was performed after pre-phasing using Shapeit (v1.r532) followed by imputation using IMPUTE2 with the 1000 Genomes Phase I integrated variant set (March 2012) as the reference panels.[^16^](#_ENREF_16)^,^[^17^](#_ENREF_17) SNPs used for imputation were restricted to those with MAF >0.5%, missing data per SNP < 5%, and Hardy-Weinberg equilibrium (HWE) >0.00001. Genetic principal components were generated using EIGENSTRAT with SNP restricting to those with MAF ≥10%, missing data per SNP < 0.5%, and HWE p-value ≥ 0.001.[^18^](#_ENREF_18)

**References**

1 Levey, A. S. *et al.* A new equation to estimate glomerular filtration rate. *Ann Intern Med* **150**, 604-612, doi:10.7326/0003-4819-150-9-200905050-00006 (2009).

2 Evans, A. M., DeHaven, C. D., Barrett, T., Mitchell, M. & Milgram, E. Integrated, nontargeted ultrahigh performance liquid chromatography/electrospray ionization tandem mass spectrometry platform for the identification and relative quantification of the small-molecule complement of biological systems. *Anal Chem* **81**, 6656-6667, doi:10.1021/ac901536h [doi] (2009).

3 Evans, A. M., B.R., B., Liu, Q., Mitchell, M. W. & Robinson, R. J. High Resolution Mass Spectrometry Improves Data Quantity and Quality as Compared to Unit Mass Resolution Mass Spectrometry in High-Throughput Profiling Metabolomics. *Metabolomics* **4** (2014).

4 Shin, S. Y. *et al.* An atlas of genetic influences on human blood metabolites. *Nat Genet* **46**, 543-550, doi:10.1038/ng.2982 (2014).

5 Weiner, J., 3rd *et al.* Biomarkers of inflammation, immunosuppression and stress with active disease are revealed by metabolomic profiling of tuberculosis patients. *PLoS One* **7**, e40221, doi:10.1371/journal.pone.0040221 (2012).

6 Hiltunen, T. P., Rimpela, J. M., Mohney, R. P., Stirdivant, S. M. & Kontula, K. K. Effects of four different antihypertensive drugs on plasma metabolomic profiles in patients with essential hypertension. *PLoS One* **12**, e0187729, doi:10.1371/journal.pone.0187729 (2017).

7 DeHaven, C. D., Evans, A. M., Dai, H. & Lawton, K. A. Organization of GC/MS and LC/MS metabolomics data into chemical libraries. *Journal of Cheminformatics* **2**, 9, doi:10.1186/1758-2946-2-9 (2010).

8 Jump, R. L. *et al.* Metabolomics analysis identifies intestinal microbiota-derived biomarkers of colonization resistance in clindamycin-treated mice. *PLoS One* **9**, e101267, doi:10.1371/journal.pone.0101267 (2014).

9 Sumner, L. W. *et al.* Proposed minimum reporting standards for chemical analysis Chemical Analysis Working Group (CAWG) Metabolomics Standards Initiative (MSI). *Metabolomics* **3**, 211-221, doi:10.1007/s11306-007-0082-2 [doi] (2007).

10 Schrimpe-Rutledge, A. C., Codreanu, S. G., Sherrod, S. D. & McLean, J. A. Untargeted Metabolomics Strategies-Challenges and Emerging Directions. *J Am Soc Mass Spectrom* **27**, 1897-1905, doi:10.1007/s13361-016-1469-y (2016).

11 Dieterle, F., Ross, A., Schlotterbeck, G. & Senn, H. Probabilistic quotient normalization as robust method to account for dilution of complex biological mixtures. Application in 1H NMR metabonomics. *Anal Chem* **78**, 4281-4290, doi:10.1021/ac051632c [doi] (2006).

12 Do, K. T. *et al.* Characterization of missing values in untargeted MS-based metabolomics data and evaluation of missing data handling strategies. *Metabolomics* **14**, 128, doi:10.1007/s11306-018-1420-2 [doi] (2018).

13 Cornelis, M. C. *et al.* The Gene, Environment Association Studies consortium (GENEVA): maximizing the knowledge obtained from GWAS by collaboration across studies of multiple conditions. *Genet Epidemiol* **34**, 364-372, doi:10.1002/gepi.20492 [doi] (2010).

14 Laurie, C. C. *et al.* Quality control and quality assurance in genotypic data for genome-wide association studies. *Genet Epidemiol* **34**, 591-602, doi:10.1002/gepi.20516 [doi] (2010).

15 Purcell, S. *et al.* PLINK: a tool set for whole-genome association and population-based linkage analyses. *Am J Hum Genet* **81**, 559-575, doi:10.1086/519795 (2007).

16 Delaneau, O., Marchini, J. & Zagury, J. F. A linear complexity phasing method for thousands of genomes. *Nat Methods* **9**, 179-181, doi:10.1038/nmeth.1785 (2011).

17 Genomes Project, C. *et al.* An integrated map of genetic variation from 1,092 human genomes. *Nature* **491**, 56-65, doi:10.1038/nature11632 (2012).

18 Price, A. L. *et al.* Principal components analysis corrects for stratification in genome-wide association studies. *Nat Genet* **38**, 904-909, doi:10.1038/ng1847 (2006).

**Supplementary Note: List of GCKD Participating Institutions and Investigators**

The nine GCKD participating institutions are: RWTH Aachen University, Aachen, Charité – University-Medicine, Berlin, Friedrich-Alexander University, Erlangen, Albert-Ludwigs-University, Freiburg, Friedrich-Schiller University, Jena, Hannover Medical School, Hannover, Medical Faculty, Ruprecht-Karls University, Heidelberg, Medical Faculty, Ludwig-Maximilians-University, Munich, and Julius-Maximilians-University, Würzburg.

A list of nephrologists currently collaborating with the GCKD study is available at <http://www.gckd.org>.

- University of Erlangen-Nürnberg

Kai-Uwe Eckardt, Heike Meiselbach, Markus P. Schneider, Mario Schiffer, Hans-Ulrich Prokosch, Barbara Bärthlein, Andreas Beck, Detlef Kraska, André Reis, Arif B. Ekici, Susanne Becker, Dinah Becker-Grosspitsch, Ulrike Alberth-Schmidt, Birgit Hausknecht, Anke Weigel

- University of Freiburg

Gerd Walz, Anna Köttgen, Ulla T. Schultheiß, Fruzsina Kotsis, Simone Meder, Erna Mitsch, Ursula Reinhard

- RWTH Aachen University

Jürgen Floege, Georg Schlieper, Turgay Saritas

- Charité, University Medicine Berlin

Elke Schaeffner, Seema Baid-Agrawal, Kerstin Theisen

- Hannover Medical School

Hermann Haller, Jan Menne, Rosemarie Hanna

- University of Heidelberg

Martin Zeier, Claudia Sommerer, Johanna Theilinger

- University of Jena

Gunter Wolf, Martin Busch, Rainer Paul

- Ludwig-Maximilians University of München

Thomas Sitter

- University of Würzburg

Christoph Wanner, Vera Krane, Antje Börner-Klein, Britta Bauer

- Medical University of Innsbruck, Division of Genetic Epidemiology

Florian Kronenberg, Julia Raschenberger, Barbara Kollerits, Lukas Forer, Sebastian Schönherr, Hansi Weissensteiner

- University of Regensburg, Institute of Functional Genomics

Peter Oefner, Wolfram Gronwald

- Institute of Medical Biometry, Informatics and Epidemiology, Medical Faculty, University of Bonn:

Matthias Schmid, Jennifer Nadal

**Supplementary Table 1: ARIC – population characteristics**

| **Variable** | **Value** |
| --- | --- |
| N | 1433 |
| Serum 6-bromotryptophan, mean (SD) | 1.07 (0.34) |
| Age, mean (SD), year | 54.6 (5.8) |
| Male, n (%) | 663 (46.3) |
| Diabetes, n (%) | 116 (8.1) |
| Hypertension, n (%) | 445 (31.1) |
| eGFR CKD-EPI, mean (SD), mL/min/1.73m^2^ | 97.7 (14.7) |
| eGFR CKD-EPI <60, n (%) | 36 (2.5) |

**Supplementary Table 2. Sensitivity analysis of the association between urine 6-bromotryptophan and incident ESKD by categorizing urine 6-bromotryptophan levels into five groups**

|  | **Cause-specific hazard ratio (95% confidence interval)** | | | | | |
| --- | --- | --- | --- | --- | --- | --- |
| **Category:^1^** | **Undetectable** | **Quartile 1** | **Quartile 2** | **Quartile 3** | **Quartile 4** | **P-trend** |
| **Model 1** | 1.00 | 0.83 (0.57, 1.20) | 0.43 (0.27, 0.70) | 0.43 (0.27, 0.70) | 0.34 (0.20, 0.59) | 6.15E-08 |
| **Model 2** | 1.00 | 0.79 (0.55, 1.14) | 0.40 (0.24, 0.64) | 0.38 (0.23, 0.61) | 0.29 (0.17, 0.49) | 5.26E-10 |
| **Model 3** | 1.00 | 0.83 (0.57, 1.20) | 0.44 (0.27, 0.72) | 0.43 (0.26, 0.70) | 0.34 (0.20, 0.59) | 2.04E-07 |
| **Model 4** | 1.00 | 0.84 (0.58, 1.22) | 0.53 (0.33, 0.86) | 0.51 (0.31, 0.83) | 0.48 (0.28, 0.83) | 1.41E-04 |
| **Model 5** | 1.00 | 1.02 (0.70, 1.49) | 0.91 (0.55, 1.49) | 1.12 (0.67, 1.85) | 1.47 (0.83, 2.62) | 3.62E-01 |

N=4,843, 216 events of interest

**1:** Subcategories according to levels of urine 6-bromotryptophan: undetectable levels; quartile 1: range (0.135;0.553); quartile 2: range (0.553;0.840), quartile 3: range (0.840;1.230), quartile 4: range (1.230;5.310)

**Covariate adjustment:**

Model 1: unadjusted

Model 2: age + sex

Model 3: Model 2 + smoking + waist-to-hip ratio + diastolic blood pressure + prevalent diabetes + coronary heart disease + high density lipoprotein cholesterol + low density lipoprotein cholesterol + diuretic use

Model 4: Model 3 + urine albumin-to-creatinine ratio

Model 5: Model 4 + eGFR

P-trend tested for the lack of linear association and was obtained by coding the undetectable category and the four quartiles as a continuous variable.

**Supplementary Table 3. Association between urine 6-bromotryptophan and death for any reason except due to forgoing dialysis and without ESKD previously**

(competing event analysis)

|  | **Cause-specific hazard ratio (95% confidence interval)** | | |
| --- | --- | --- | --- |
| **Category:^1^** | **Undetectable** | **Low** | **High** |
| **Model 1** | 1.00 | **0.69 (0.51, 0.92)** | **0.70 (0.52, 0.93)** |
| **Model 2** | 1.00 | **0.63 (0.47, 0.84)** | **0.65 (0.48, 0.87)** |
| **Model 3** | 1.00 | 0.76 (0.57, 1.02) | 0.92 (0.68, 1.25) |
| **Model 4** | 1.00 | 0.77 (0.58, 1.04) | 0.96 (0.71, 1.30) |
| **Model 5** | 1.00 | 0.82 (0.61, 1.11) | 1.09 (0.80, 1.50) |

N=4,843, 216 events of interest and 273 competing events

**1:** Subcategories according to levels of urine 6-bromotryptophan: undetectable levels; low: range: 0.135 to <0.840(median); high: range: 0.840(median) to 5.310

**Covariate adjustment:**

Model 1: unadjusted

Model 2: age + sex

Model 3: Model 2 + smoking + waist-to-hip ratio + diastolic blood pressure + prevalent diabetes + coronary heart disease + high density lipoprotein cholesterol + low density lipoprotein cholesterol + diuretic use

Model 4: Model 3 + urine albumin-to-creatinine ratio

Model 5: Model 4 + eGFR

**Supplementary Table 4: Summary of properties of urine 6-bromotryptophan and its measurement in the GCKD study**

| **Biochemical** | **6-bromotryptophan** |
| --- | --- |
| Super-Pathway | Amino Acid |
| Sub-Pathway | Tryptophan Metabolism |
| COMP ID | 53242 |
| Platform | LC/MS Neg |
| Chemical ID | 100009338 |
| Retention Index | 3525 |
| Mass (m-H) | 280.9931 |
| CAS | 52448-17-6 |
| PubChem | 3496442 |
| ChemSpider | 2736705 |
| KEGG | - |
| HMDB ID | - |
| Relative standard deviation (RSD) | 0.11 |
| Samples without measurement, n (%) | 2179 (43%) |
| Samples with measurement, n (%) | 2909 (57%) |
| Measurements, median (range) | 0.84 (0.14-5.31) |

Upon data receipt, metabolite identifiers were PubChem ID 96735, ChemSpider ID 2736705, and no CAS was available. This information was updated before the beginning of the project.

**Supplementary Figure 1: Regional association plot of the *ERO1A* locus in the ARIC study** (N=1,433)

**
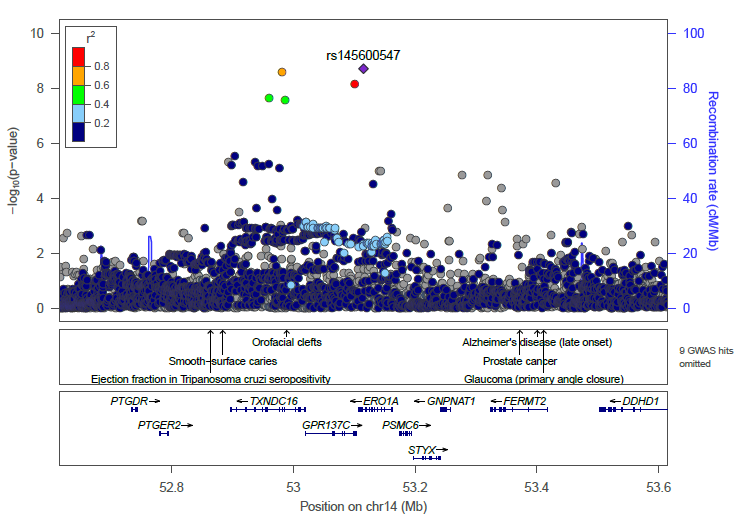
**

**Supplementary Figure 2: Distributions of urine 6-bromotryptophan measurements** (N=4,843)

**(A) Urine 6-bromotryptophan (corrected for urine dilution)**

**
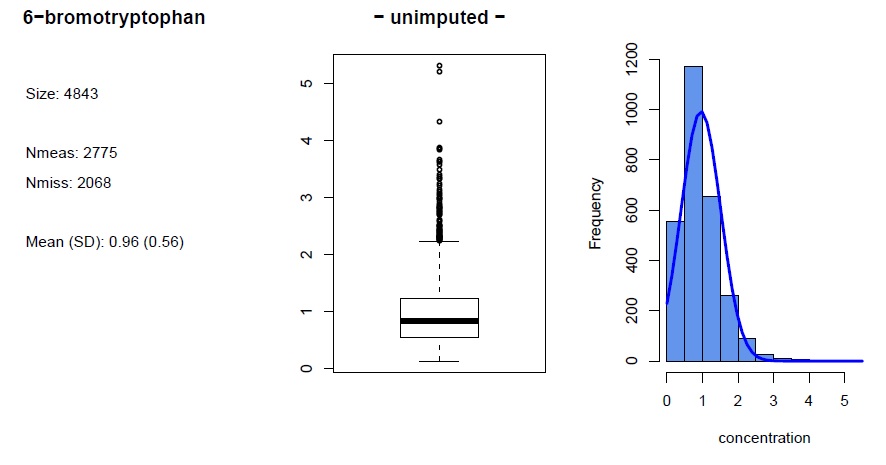
**

**(B) Urine 6-bromotryptophan (corrected for urine dilution) – across year of baseline visit**

**
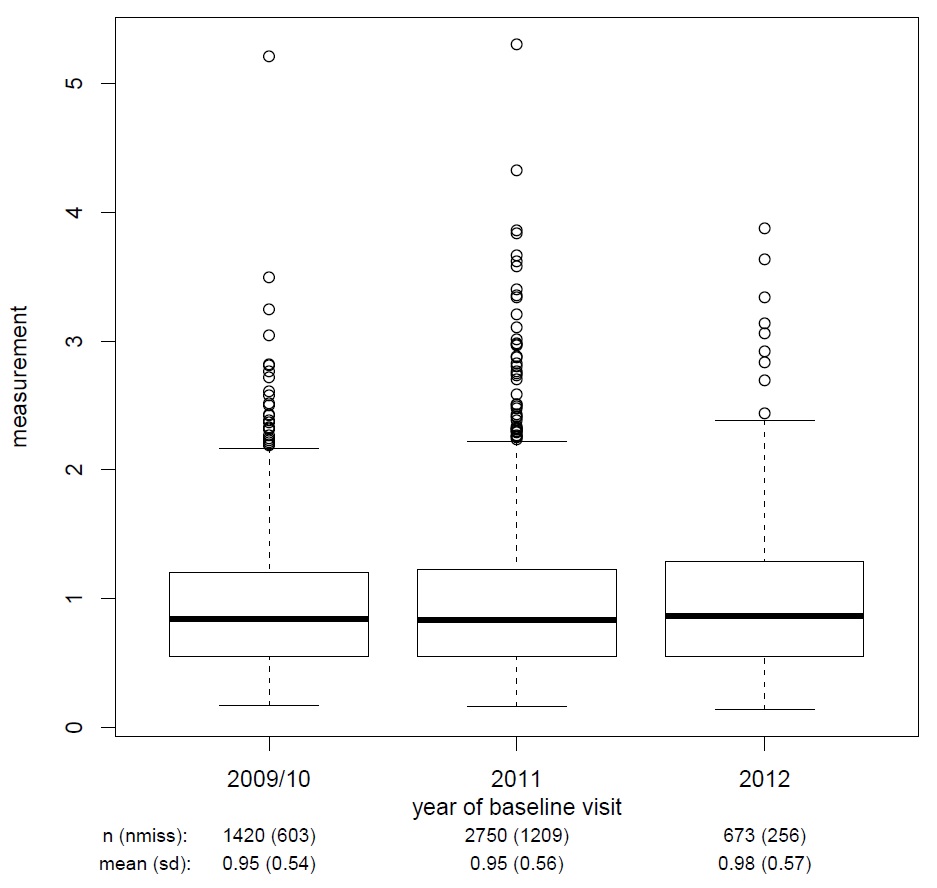
**

**(C) Urine 6-bromotryptophan (minimum-imputed, corrected for urine dilution, log_2_-transformed) – used in GWAS**

**
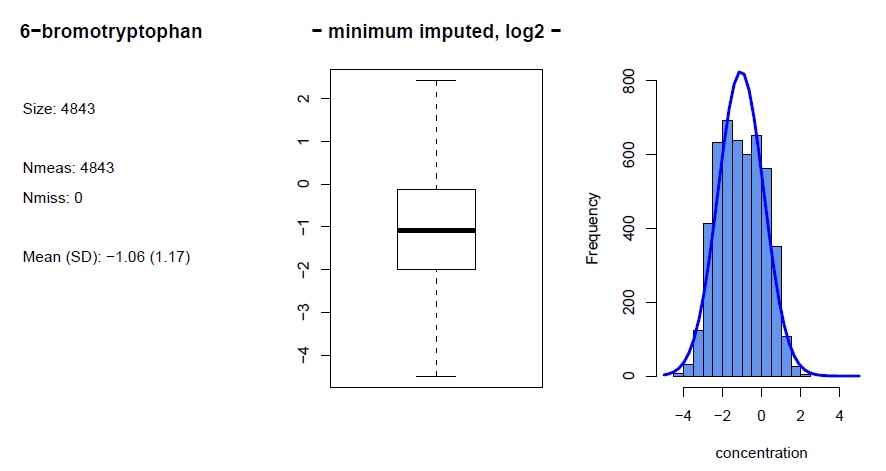
**

**(D) Urine 6-bromotryptophan (3 categories) – used in cross-sectional and prospective analyses**

**
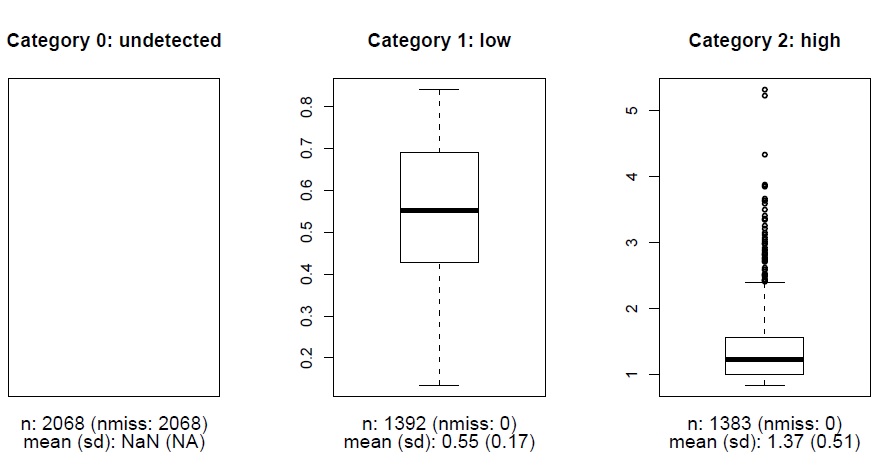
**

Subcategories according to levels of 6-bromotryptophan (corrected for urine dilution): 0, undetectable levels; 1, Range: 0.135 to <0.840(median); 2, Range: 0.840(median) to 5.310

**Supplementary Figure 3:** **Q-Q plot and Manhattan plot of the GWAS of urine 6-bromotryptophan in the GCKD cohort** (N=4,911)

**(A) Q-Q plot**


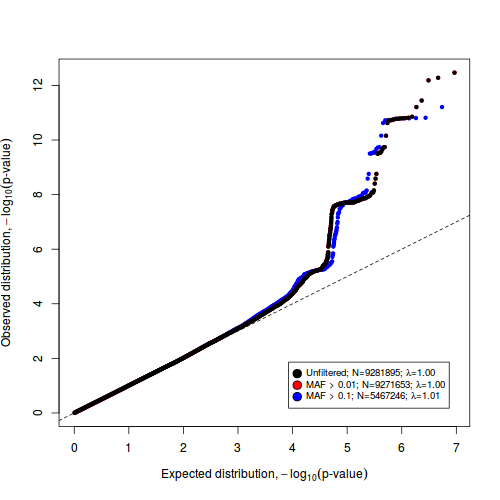


**(B) Manhattan plot**


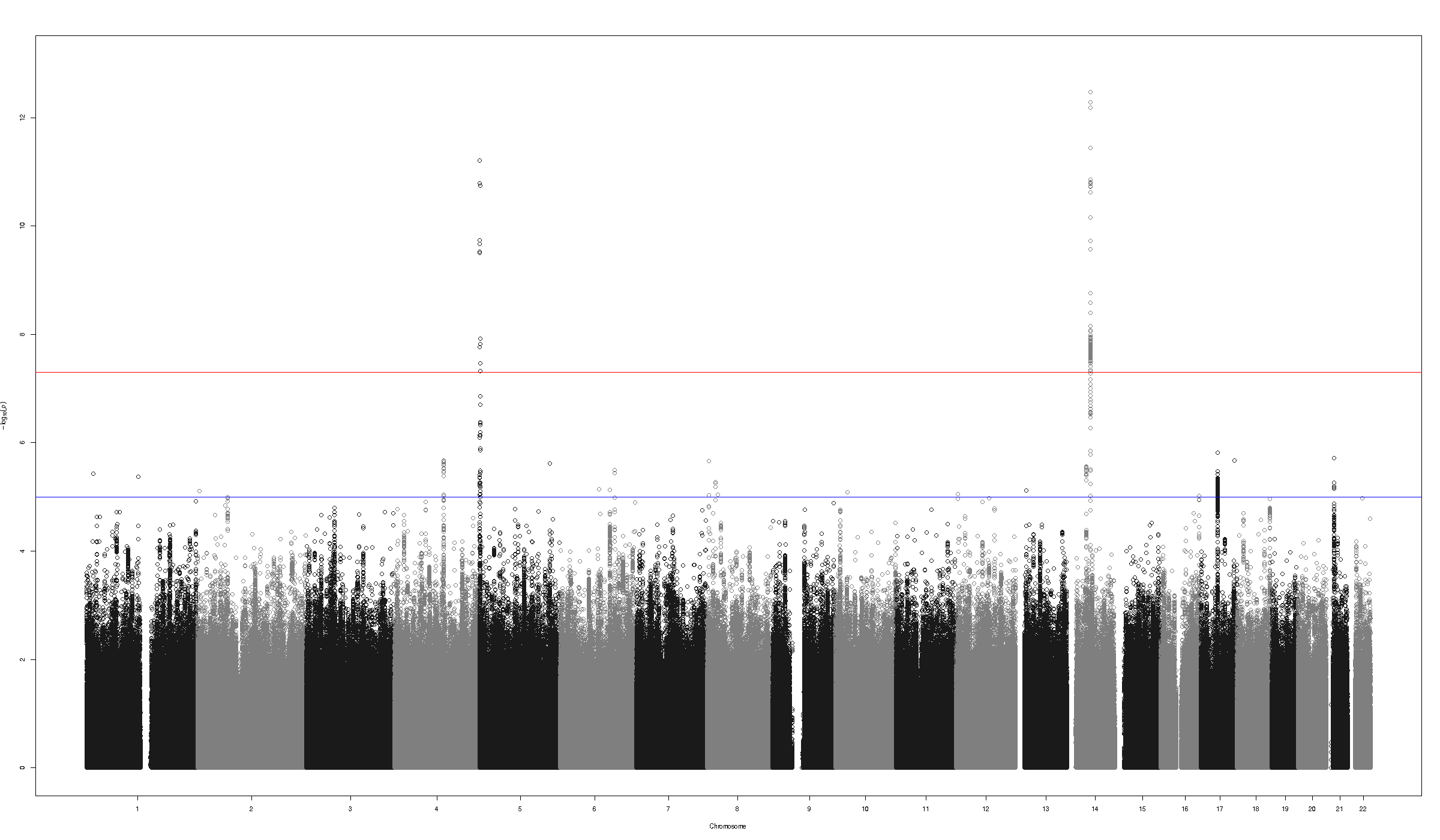


1. The genomic control parameter (lambda) of 1.0 indicates no inflation, consistent with the absence of confounding by unmodeled population substructure. The plot was generated with the R package *gwasqc*.
2. Manhattan plot shows the two significant loci on chromosomes 5 and 14. X axis: chromosomal position, Y axis: -log_10_(p-value) for association.

**Supplementary Figure 4**. **Plots of urine 6-bromotryptophan levels in relation to baseline kidney function markers** (N=4,843)

**(A) Three categories of urine 6-bromotryptophan used in univariate and primary prospective analysis**

**
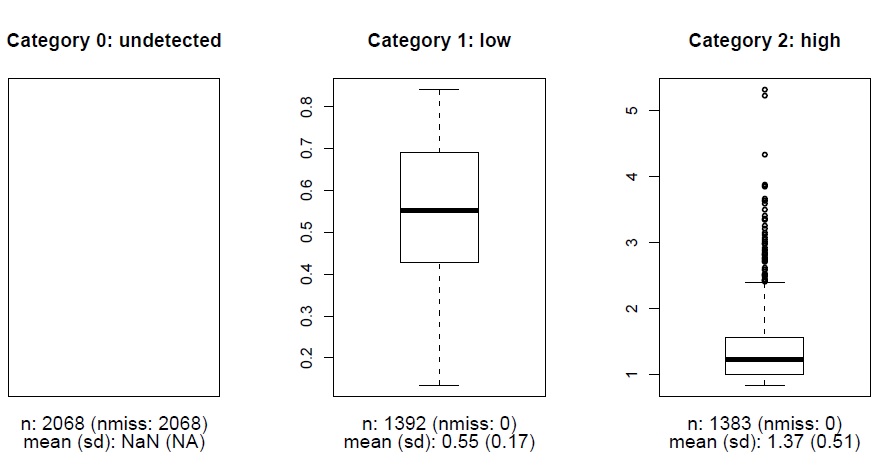
**

**(B) Box and scatter plots of eGFR versus urine 6-bromotryptophan levels**


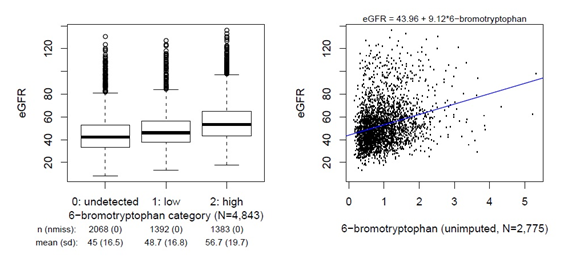


**(C) Box and scatter plots of UACR versus urine 6-bromotryptophan levels**


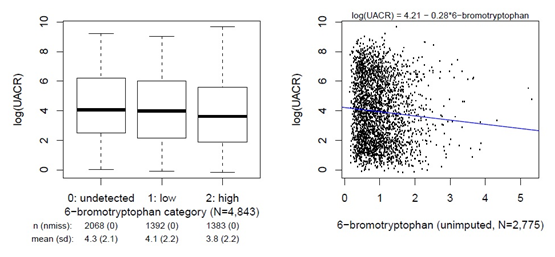

Supplement: Supplementary file 1 — Supplementary information. [file 41598_2020_66334_MOESM1_ESM.docx]
